# Insomnia among college students: A bibliometric analysis from 2003 to 2022

**DOI:** 10.1097/MD.0000000000038227

**Published:** 2024-05-17

**Authors:** Mengdie Yang, Lingling Li

**Affiliations:** a Student Affairs Office, Hebei University of Chinese Medicine, Shijiazhuang, Hebei, China; b School of Rehabilitation Science, Shanghai University of Traditional Chinese Medicine, Shanghai, China.

**Keywords:** bibliometric analysis, CiteSpace, college students, insomnia, VOSviewer

## Abstract

**Background:**

Insomnia has become a common health problem in modern society, especially among college students. The purpose of this study was to analyze the research status, research hotspots and frontier trends of insomnia among college students over the past 20 years.

**Methods:**

VOSviewer 1.6.19 and CiteSpace 6.2 were used. R4: This study conducts a bibliometric and visualization analysis of the annual publications, authors, countries/regions, institutions, categories, journal/literature cocitations and keywords related to insomnia among college students in the Web of Science (WoS) core collection from 2003 to 2022.

**Results:**

A total of 590 studies were included, and the number of studies on insomnia among college students has steadily increased over the last 20 years. The authors of high yield are represented by Taylor DJ and Miller MB. The countries/regions with high yields were the USA and China. The institutions of high yield were King Saud University and Southern Medical University. Its research fields were mainly Clinical Neurology, Psychiatry and Neurosciences. Mental health and insomnia, sleep quality and the impact of coronavirus disease 2019 (COVID-19) on insomnia are current research hotspots. Future research could focus on predicting the chronotype and physical activity of insomnia students.

**Conclusion:**

Through bibliometric and visualization analysis, this study investigated insomnia among college students over the past 20 years and preliminarily revealed the findings of coauthors and institutions. This study provides a general understanding of the research hotspots and frontier trends of insomnia among college students and provides some references for future research.

## 1. Introduction

Sleep disorders are a type of health condition in which individuals experience problems falling asleep, maintaining sleep, or obtaining sleep of adequate quality and are usually characterized by difficulty falling asleep, reduced sleep quality, and reduced sleep time. Insomnia, one of the most common sleep disorders, is characterized by dissatisfaction with sleep duration or quality, difficulty initiating or maintaining sleep, severe distress and daytime dysfunction.^[1]^

Insomnia is not only widespread among the general population but also particularly prominent among college students. The college student period, as an important stage in an individual life, is often accompanied by academic pressure, social pressure and enormous changes in lifestyle. The particularity of this stage of life makes college students more susceptible to sleep problems such as insomnia. Insomnia is not just a superficial sleep disturbance; it also has a profound impact on college students’ physical and mental health and daily life. Among college students, insomnia can lead to a range of adverse consequences, including but not limited to inattention in class, decreased learning efficiency, mood swings, increased depression and anxiety, strained social relationships, and increased physical health problems. These problems not only pose a serious threat to the quality of life of individual college students but also may affect their future career and social development. Research shows that the incidence of insomnia among college students has a steady upward trend, reaching as high as 13.0% to 30.3%.^[2]^ That is, insomnia damages college students’ bodies and psychology, affects many aspects of their learning and life functions, and greatly reduces their happiness and satisfaction in life.^[3]^

Bibliometrics was originally proposed by the British intelligence scientist Pritchard as a method of mapping, measuring, monitoring and studying scientific results in a specific field.^[4]^ CiteSpace and VOSviewer are more commonly used knowledge graph visual analysis tools. The former is software developed by Drexel University Dr Chaomei Chen team based on the JAVA language. It mainly conducts quantitative analysis of the literature in specific fields to explore the key areas, paths and focuses of knowledge evolution and can analyze the evolution of a knowledge field. The process is centrally displayed in the network graph.^[5]^ The latter is a bibliometric analysis software jointly developed by scholars Nees Jan van Eck and Ludo Waltman of Leiden University in the Netherlands for drawing knowledge maps,^[6]^ such as cocitation analysis, cooccurring word analysis and document coupling analysis.^[7]^ The distance and correlation strength between elements reflect their characteristics and are displayed visually.^[7]^

Considering that there is currently no bibliometric analysis of college student insomnia that systematically summarizes this field, to gain a deeper understanding of the evolving trends, influencing factors, and possible intervention strategies for this topic, we need to conduct a detailed bibliometric analysis. This study used the Web of Science (WoS) Core Collection as the data source, VOSviewer 1.6.19 and CiteSpace 6.2. R4 To intuitively, clearly and multiperspectively sort and analyze the relevant literature published in the past 20 years and explore the research hot spots and development of insomnia among college students. Trends and future directions. Thus, we can better understand the overall picture of insomnia among college students, provide a strong scientific basis for developing targeted intervention measures, help college students overcome insomnia problems, and improve their quality of life and academic achievements.

## 2. Materials and methods

### 2.1. Data collection and search strategies

The related literature on insomnia among college students was searched in the WoS core collection. The search strategies used were TS = (college student OR university student OR undergraduate student OR master student OR doctoral student OR PhD student OR undergraduate student OR postsecondary education OR undergraduate tertiary education OR undergraduate) AND (insomnia OR insomnia disorder OR sleep initiation and maintenance disorder OR disorder of initiating and maintaining sleep OR early awakening OR primary insomnia OR sleeplessness OR transient insomnia OR chronic insomnia OR secondary insomnia OR sleep-wake disorder OR sleep initiation dysfunction). The publication language was English, and the publication years ranged from January 1, 2003, to December 31, 2022. Only articles and reviews were included; meeting abstracts, letters, book chapters and obviously irrelevant literature were excluded. The search queries used are listed in Table 1.

**Table 1:** The search queries.

Set	Results	Search query
#1	95120	TS = (college student OR university student OR undergraduate student OR master student OR doctoral student OR PhD student OR undergrad student OR postsecondary education OR undergraduate tertiary education OR undergraduates) AND LA = (English) AND DT = (Article OR Review) AND PY = (2003–2022)
#2	27601	TS = (insomnia OR insomnia disorder OR sleep initiation and maintenance disorder OR disorder of initiating and maintaining sleep OR early awakening OR primary insomnia OR sleeplessness OR transient insomnia OR chronic insomnia OR secondary insomnia OR sleep-wake disorder OR sleep initiation dysfunction) AND LA = (English) AND DT = (Article OR Review) AND PY = (2003–2022)
#3	590	#1 AND #2

### 2.2. Bibliometric analysis and visualization

The above 590 included studies were named Download_XX.txt, downloaded, and saved as full records and cited references in plain text format. Given the complexity and vastness of the dataset, we employed advanced bibliometric and visualization analysis tools, namely VOSviewer 1.6.19 and CiteSpace 6.2.R4. These tools are widely recognized in the field for their ability to handle large volumes of scholarly data and to generate insightful visualizations that facilitate the exploration of research trends, collaborations, and hotspots. By utilizing these tools, we were able to conduct comprehensive analyses of various aspects of the collected literature, including annual publications; authors; countries/regions; institutions; categories; journal/literature cocitations; and keywords. This approach enabled us to gain a deeper understanding of the research landscape surrounding college students’ insomnia and to identify key patterns and trends within the literature. This study did not need ethical approval because this article is a bibliometric analysis without the participation of patients or animals. This paper will be submitted to experts in this field for peer review and widely disseminated at home and abroad.

## 3. Results

### 3.1. Annual publications and citations

**Figure 1.**
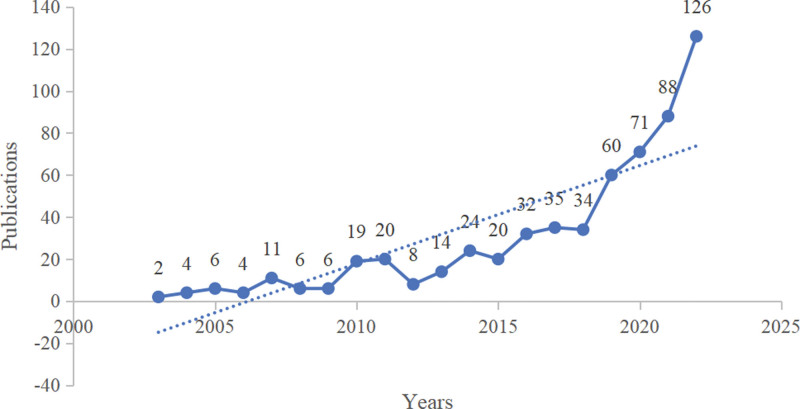
Annual publication trend chart.

**Figure 2.**
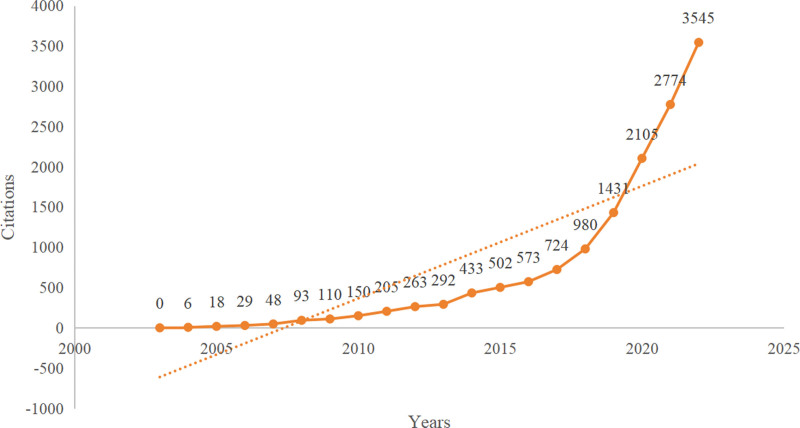
Citations of annual publications.

A total of 590 studies were included. From 2003 to 2022, the average number of publications per year was approximately 30, and the number of annual publications showed an overall upward trend (Fig. 1), reaching its peak in 2022 (126). At present, research on insomnia among college students is in the rapid development stage and has good prospects. In addition, the number of paper citations has shown a continuing growth trend (Fig. 2).

### 3.2. Coauthor analysis

**Figure 3.**
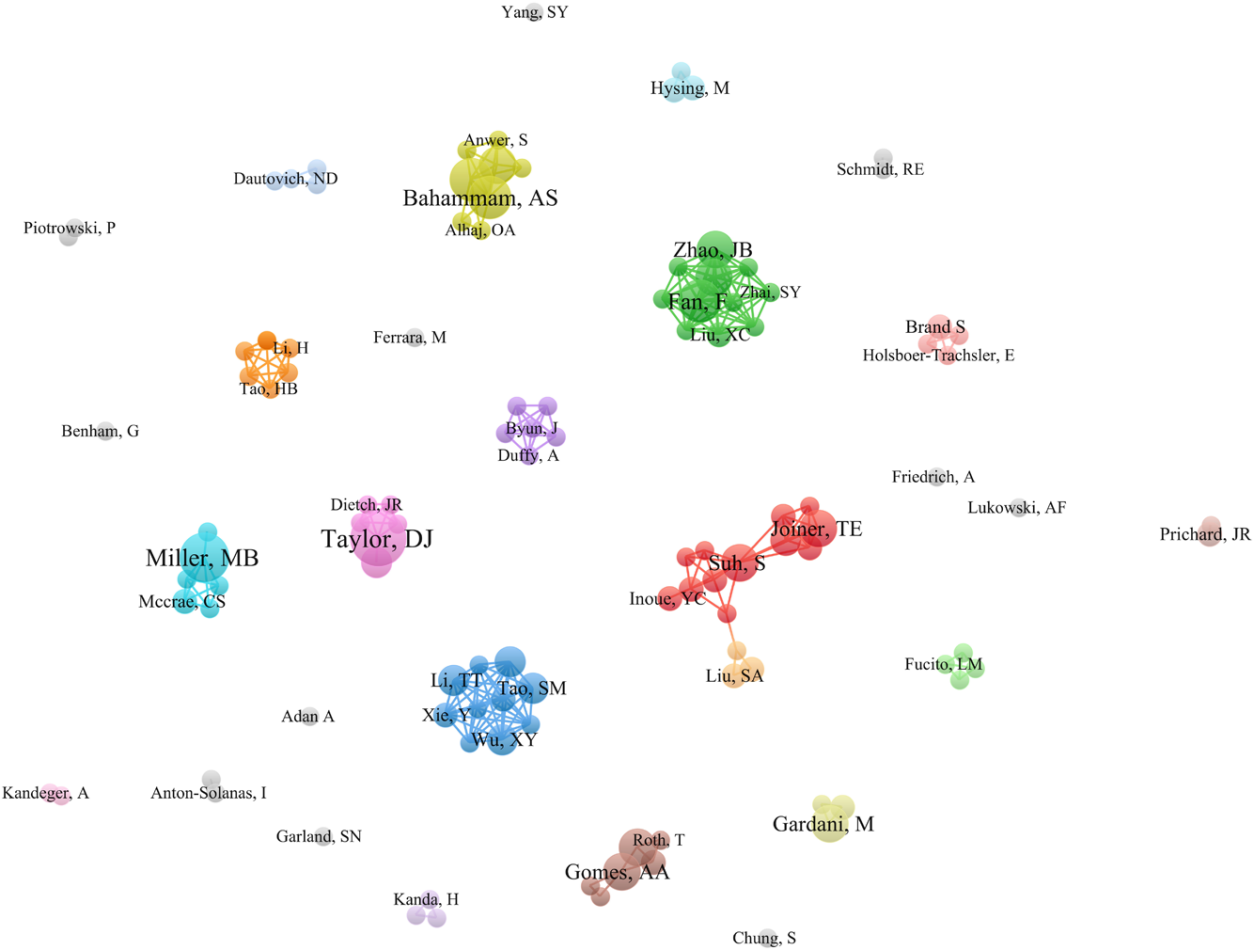
Author collaboration network.

The authors were used as nodes for analysis, and the visualized chart is shown in Figure 3. The top 5 authors were Taylor DJ (9), Miller MB (8), Bahammam AS (7), Fan F (7) and Pandi-Perumal SR (7), followed by Gardani M (6), Gomes AA (6), Suh S (6) and Zhao JB (6).

### 3.3. Countries/regions analysis

**Figure 4.**
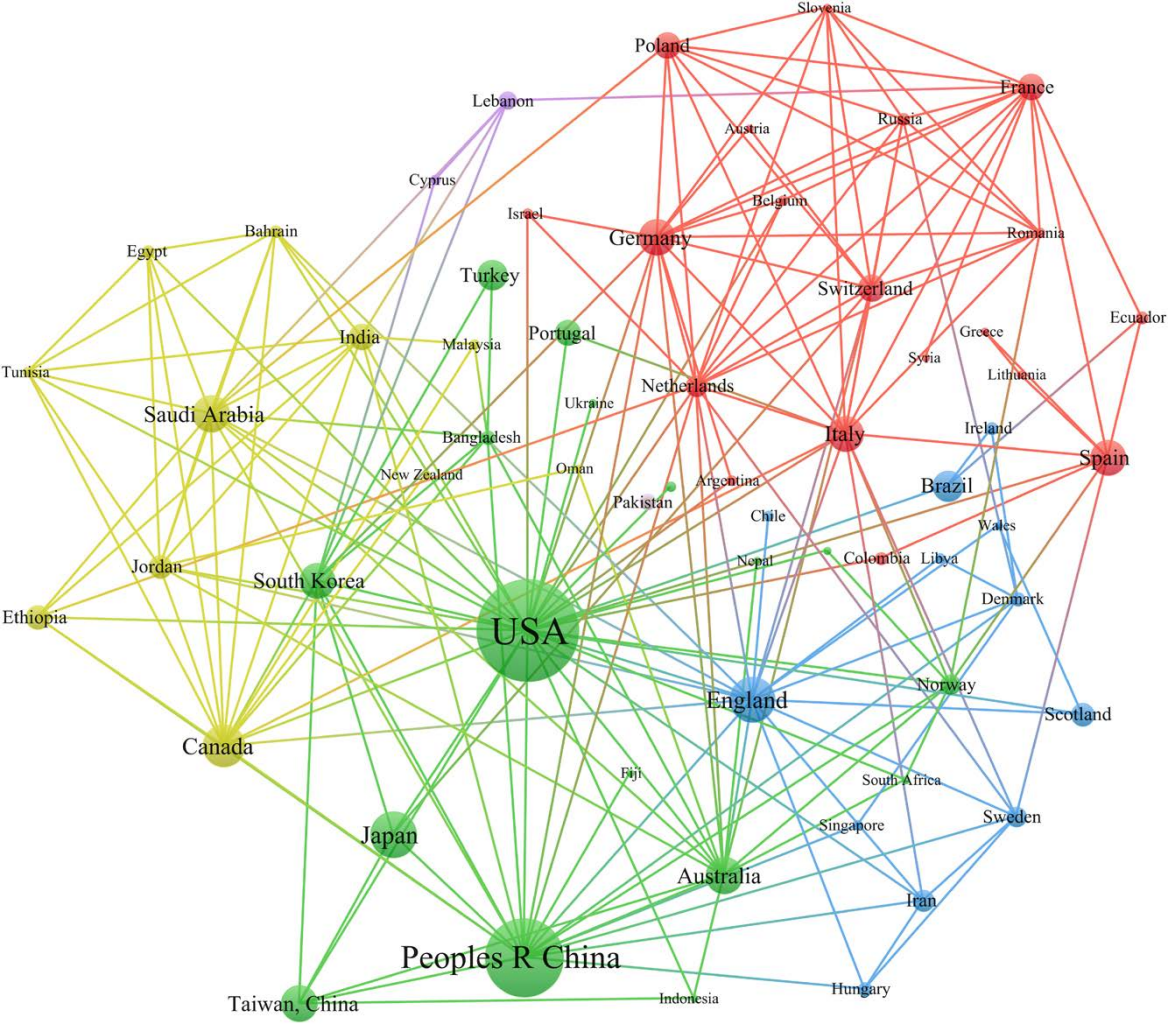
Collaboration network of countries and regions.

The countries/regions were used as nodes for analysis, and the visualized chart is shown in Figure 4. The top 5 countries/regions were the USA (184), China (109), Japan (39), England (38) and Canada (33), followed by Australia (25) and Saudi Arabia (25).

### 3.4. Institutions analysis

**Figure 5.**
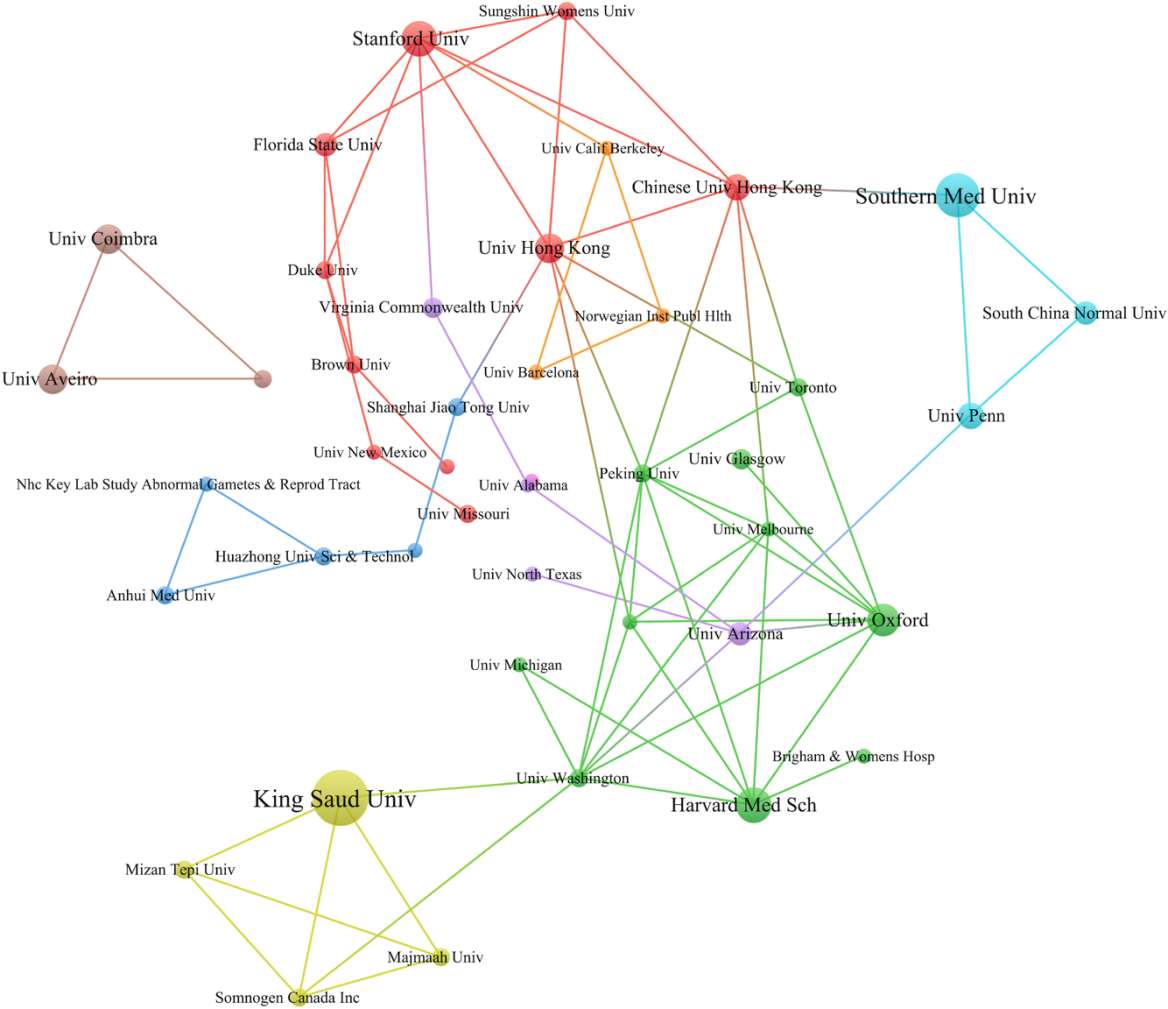
Collaboration network of institutions.

The institutions were used as nodes for analysis, and the visualization chart is shown in Figure 5. The top 5 institutions were King Saud University (19), Southern Medical University (15), Harvard Medical School (12), Stanford University (11) and the University of Oxford (11), followed by the University of Aveiro (10), the University of Coimbra (10) and the University of Hong Kong (10).

### 3.5. Analysis of WoS categories

There were 71 WoS categories in the included studies, the top 20 of which are shown in Table 2. The top 5 WoS categories were Clinical Neurology (187), Psychiatry (162), Neurosciences (96), Public Environmental Occupational Health (81) and Environmental Sciences (44), followed by Medicine General Internal (31), Nursing (28) and Health Care Sciences Services (26).

**Table 2:** Top 20 Web of Science categories.

Web of Science categories	Frequency	Percentage (%)
Clinical Neurology	187	31.70
Psychiatry	162	27.46
Neurosciences	96	16.27
Public Environmental Occupational Health	81	13.73
Environmental Sciences	44	7.46
Medicine General Internal	31	5.25
Nursing	28	4.75
Health Care Sciences Services	26	4.41
Physiology	26	4.41
Biology	25	4.24
Multidisciplinary Sciences	23	3.90
Psychology Clinical	21	3.56
Health Policy Services	16	2.71
Psychology	15	2.54
Respiratory System	14	2.37
Substance Abuse	14	2.37
Medicine Research Experimental	13	2.20
Integrative Complementary Medicine	8	1.36
Behavioral Sciences	7	1.19
Psychology Biological	7	1.19

### 3.6. Analysis of cited references

A total of 554 references were cited, the top 10 of which are listed in Table 3. The top 5 cited studies were Lund HG, 2010 (955), Wolfson AR, 2003 (454), Marelli S, 2021 (344), Freeman D, 2017 (337) and Mah CD, 2011 (321), followed by Majumdar P, 2020 (209), Younes F, 2016 (199) and Nadorff MR, 2011 (191).

**Table 3:** Top 10 cited studies.

Title	First author	Yr	Cited frequency	Journal	DOI
Sleep patterns and predictors of disturbed sleep in a large population of college students^[8]^	Lund HG	2010	955	J Adolesc Health	10.1016/j.jadohealth.2009.06.016
Understanding adolescents’ sleep patterns and school performance: A critical appraisal^[9]^	Wolfson AR	2003	454	Sleep Med Rev	10.1016/s1087-0792(03)90003-7
Impact of COVID-19 lockdown on sleep quality in university students and administration staff^[10]^	Marelli S	2021	344	J Neurol	10.1007/s00415-020-10056-6
The effects of improving sleep on mental health (OASIS): A randomized controlled trial with mediation analysis^[11]^	Freeman D	2017	337	Lancet Psychiatry	10.1016/S2215-0366(17)30328-0
The effects of sleep extension on the athletic performance of collegiate basketball players^[12]^	Mah CD	2011	321	Sleep	10.5665/SLEEP.1132
COVID-19 pandemic and lockdown: cause of sleep disruption, depression, somatic pain, and increased screen exposure of office workers and students of India^[13]^	Majumdar P	2020	209	Chronobiol Int	10.1080/07420528.2020.1786107
Internet addiction and relationships with insomnia, anxiety, depression, stress and self-esteem in university students: A cross-sectional designed study^[14]^	Younes F	2016	199	PLoS One	10.1371/journal.pone.0161126
Insomnia symptoms, nightmares, and suicidal ideation in a college student sample^[15]^	Nadorff MR	2011	191	Sleep	10.1093/sleep/34.1.93
Sleep patterns in college students: gender and grade differences^[16]^	Tsai LL	2004	184	J Psychosom Res	10.1016/S0022-3999(03)00507-5
Daily activities and sleep quality in college students^[17]^	Carney CE	2006	164	Chronobiol Int	10.1080/07420520600650695

COVID-19 = coronavirus disease 2019.

### 3.7. Analysis of cited journals

**Figure 6.**
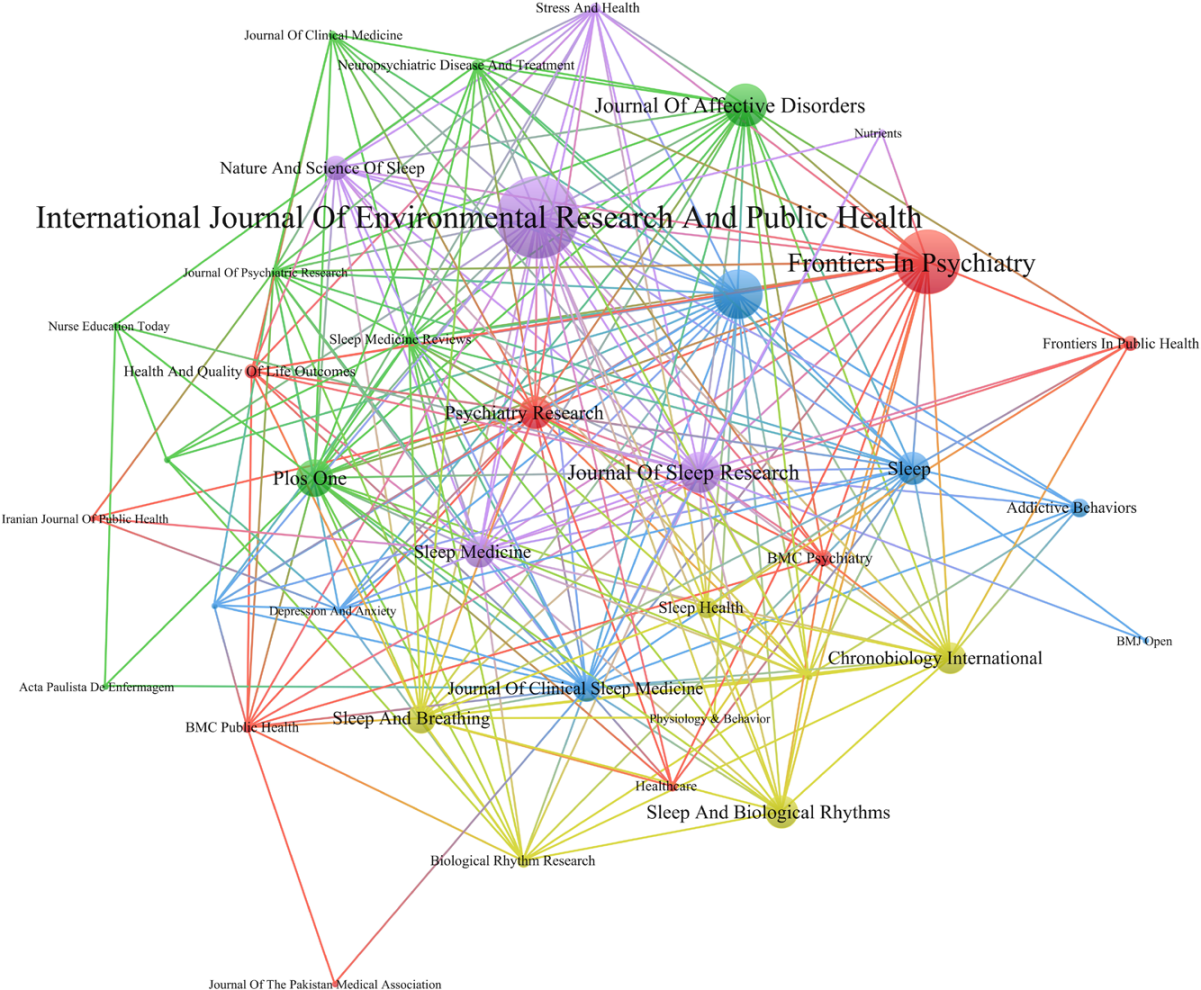
Journal cocitation network.

The journal cocitations were used as nodes for analysis, and the visualized chart is shown in Figure 6. The top 5 journal cocitations were International Journal of Environmental Research and Public Health (40), Frontiers in Psychiatry (31), Behavioral Sleep Medicine (24), Journal of Affective Disorders (21) and Journal of Sleep Research (20), followed by PLoS One (18), Sleep (16), Sleep and Biological Rhythm (16) and Psychiatry Research (16).

### 3.8. Analysis of cooccurring keywords

**Figure 7.**
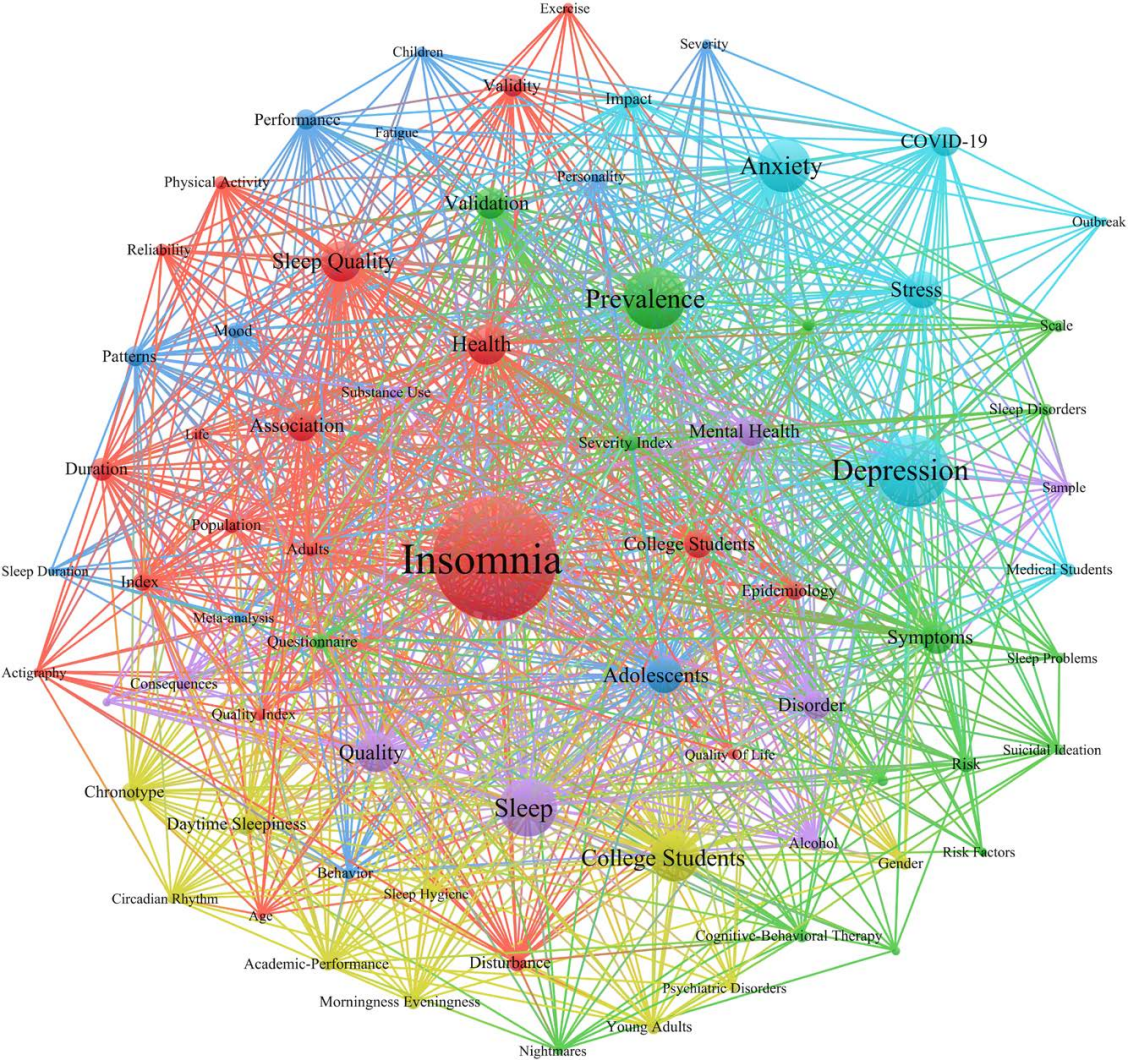
Keyword co-occurrence network.

The keywords were used as nodes for analysis, and the visualization is shown in Figure 7. The high-frequency keywords (frequency ≥ 80) used were insomnia (339), depression (180), prevalence (149), sleep (138), anxiety (126), college students (114), health (91), sleep quality (91), quality (87), stress (83) and adolescents (81).

### 3.9. Analysis of keyword clusters

**Figure 8.**
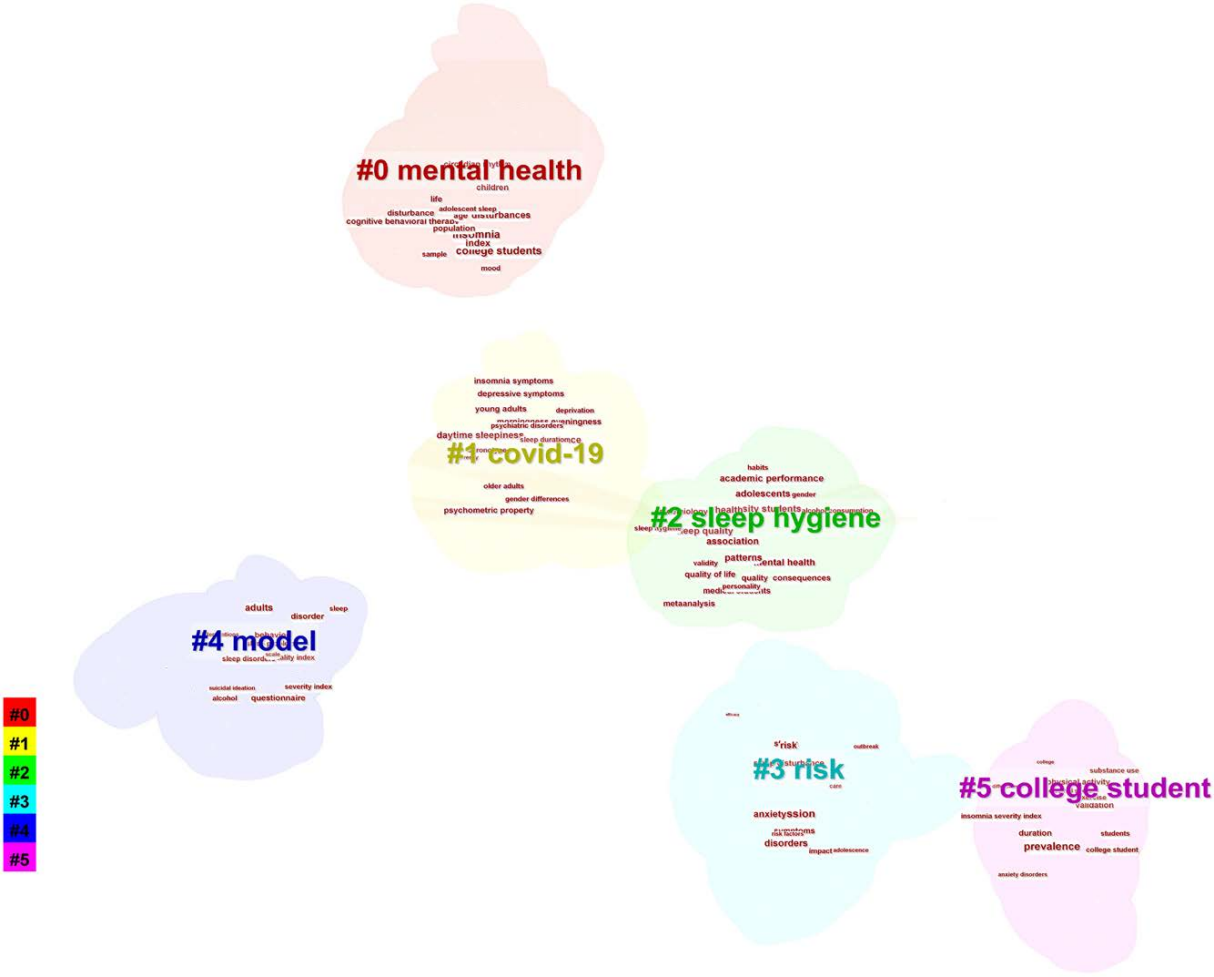
Keyword clustering chart.

The LLR algorithm was used to cluster the keywords, and 6 clusters were formed (Fig. 8), namely, mental health, coronavirus disease 2019 (COVID-19), sleep hygiene, risk, model and college student.

### 3.10. Analysis of the strongest burst keywords

**Figure 9.**
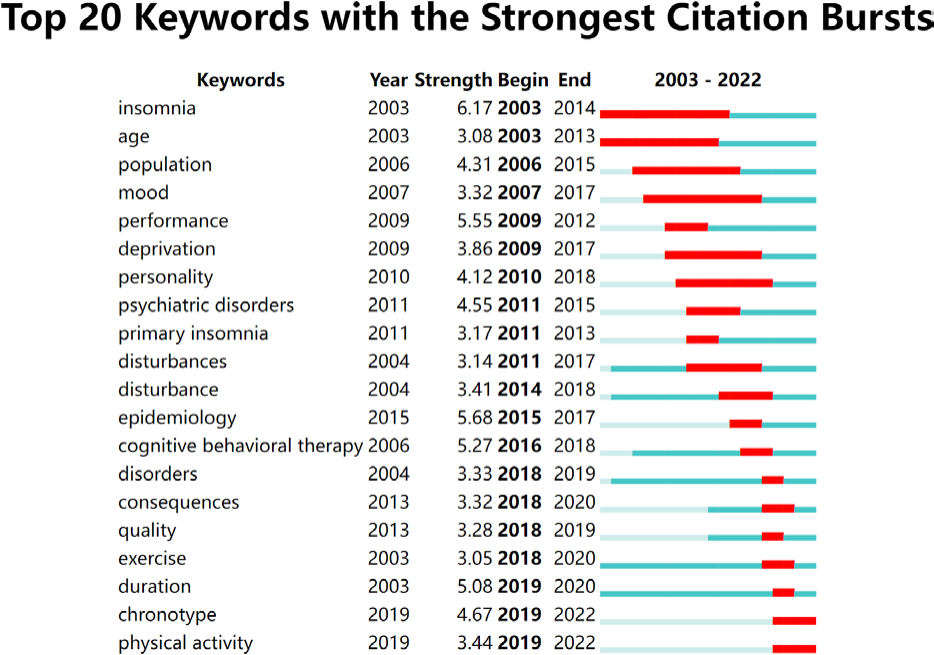
Top 20 keywords with the strongest citation bursts.

The burst algorithm was used to predict the frontier trend, and the burst keywords included chronotype and physical activity (Fig. 9).

## 4. Discussion

This study uses the relevant literature on college students’ insomnia in the WoS core collection from the past 20 years as the data source and uses VOSviewer 1.6.19 and CiteSpace 6.2. R4 for data analysis systematically analyzes the field and draws the corresponding knowledge map, which is relatively intuitive. This paper comprehensively presents an overview of college student insomnia research over the past 20 years, preliminarily reveals the collaboration of researchers and institutions, and provides a general understanding of the research hot spots, frontiers and research trends related to college student insomnia. These findings can guide future topic selection and research on college student insomnia. This study provides a reference for future research directions.

The number of documents on a subject in a specific period can not only reflect its development trend but also reflect the importance researchers attach to this field.^[18]^ Judging from the number of annual publications, the number of annual publications on insomnia among college students is generally on the rise, and the number of papers published in the next few years will continue to increase, which may be related to people paying more attention to the physical and mental health of college students. From 2003 to 2022, the number of publications ranged from 2 to 126. Moreover, the number of citations in the literature has also increased, which shows that research on insomnia among college students is gradually deepening and gradually gaining more recognition and attention.

According to the author cooperation network, the teams represented by Taylor DJ, Miller MB, Bahammam AS, Fan F, and Pandi-Perumal SR have published a high number of papers, but the cooperation network among the authors is sparse. For example, Taylor DJ focused on the relationship between college students’ insomnia and their studies and emotions, while Miller MB focused on the relationship between insomnia and drinking. From the perspective of the institutional cooperation network, the institutions associated with insomnia among college students are mainly universities, such as King Saud University but also some hospitals and scientific research institutions, and the cooperation among teams and institutions is also relatively close. From the perspective of national/regional cooperation networks, there are some regional differences in the total number of publications. The number of publications in economically developed countries is much greater than that in countries with poor economic levels, which may be related to poor scientific research conditions and weak scientific research awareness in economically underdeveloped countries. Moreover, the development level of scientific research is closely related to the economy.^[19]^ The USA has the largest number of publications, far exceeding that of other countries, which also shows that the USA has a high contribution rate to this field and a great influence on publications. Second, China and Japan are the few productive Asian countries. It is suggested that different authors, institutions and countries/regions carry out more relevant academic exchange activities in the future, strengthen academic exchange and cooperation, jointly improve the overall strength of scientific research in this field, and promote the rapid development of insomnia among college students.

There are many categories of insomnia among college students, mainly concentrated in the field of Clinical Neurology, Psychiatry and Neurosciences. According to the journal cocitation cooperation network, the International Journal of Environmental Research and Public Health and Frontiers in Psychiatry are influential journals in this field; these journals involve research on mental illness, predictive imaging and genomics, as well as computational modeling and novel biomarkers, laying the foundation for an integrated biosocial context in public mental health, social well-being and cutting-edge clinical practice and specialization. The overall impact factors of several highly cited journals are greater. That is, related research on insomnia among college students has gradually attracted the attention of many scholars, and it has research value.

Keywords are words or terms extracted from titles, abstracts or texts that can express the topic of literature research and have retrieval significance. If a keyword appears many times in the literature of its own field, the research topic represented by the keyword is considered to be the research hotspot and focus in that field.^[20]^ The co-occurrence of keywords refers to the phenomenon in which 2 or more keywords appear in a study at the same time. We performed a visualization analysis of 590 studies on keywords to explore the research hotspots of insomnia among college students. The size of the nodes in the visualized chart indicates the frequency of the keywords.^[21]^ The greater the number of cooccurrences there are, that is, the thicker the connection between 2 nodes is, the closer the theme content represented by the keyword is. Combined with keyword co-occurrence and keyword cluster analysis, the research hotspots can be roughly divided into the following research on the relationships between college students’ mental health and insomnia and between sleep quality and the impact of COVID-19 on insomnia.

Research on the relationship between college students’ mental health and insomnia has involved mainly depression, anxiety, stress, etc. This topic has attracted widespread attention and accounts for half of the 10 most cited documents. Insomnia is not only a common accompanying symptom of depression and anxiety but also may be mutually reinforcing. That is, insomnia may worsen symptoms of depression and anxiety, and depression and anxiety may worsen insomnia. The results of a single-blind, randomized controlled trial by Freeman showed that difficulty sleeping is a contributing factor to the occurrence of mental health problems and that improving sleep is beneficial to mental health.^[11]^ College students usually face pressure from many means, such as academics, society and daily life. These stresses can be closely linked to insomnia, creating a vicious cycle. High levels of stress may lead to insomnia, and insomnia may exacerbate individual feelings of stress.

The problem of insomnia among college students in the context of the COVID-19 epidemic is a hot topic of research for Chinese scholars. Figure 8 #1 shows that a cluster was formed in this field. The COVID-19 epidemic may be a serious stressor for college students and have a negative impact on their physical and mental health. The COVID-19 pandemic is not just a health crisis; it is also a psychological crisis. College students are particularly vulnerable, as they may face academic stress, social isolation, and concerns about employment prospects. These factors may have been further amplified during the pandemic, causing college students to experience unprecedented levels of stress and anxiety. This continued psychological stress may lead to an increase in insomnia problems. The COVID-19 pandemic has added additional challenges to mental health and insomnia issues among college students. Future research can further explore the complex relationship between insomnia and the COVID-19 pandemic to develop more targeted interventions to help college students cope with the challenges of special health events.

In our exploration of the research hotspots and future directions of insomnia among college students, we aimed to identify emerging areas of research that hold significant theoretical and practical implications. One such area is the investigation of chronotype and physical activity as potential determinants of insomnia among college students. Chronotype refers to individual differences in the timing of biological rhythms, such as sleep-wake patterns, which have been increasingly recognized as influential factors in sleep quality and mental health outcomes. Understanding how chronotype interacts with college students’ lifestyle factors, including physical activity levels, can provide valuable insights into personalized interventions for improving sleep quality and overall well-being. Moreover, as society undergoes continuous changes in lifestyle and technological advancements, the landscape of college students’ insomnia evolves accordingly. It is essential to delve deeper into these societal shifts and their impact on sleep habits and mental health among college students. By acknowledging and addressing these evolving concerns, researchers can develop more tailored and effective prevention and treatment strategies for insomnia in this population. Therefore, future research should focus on conducting more in-depth explorations of the complex relationships among college students’ lifestyles, sleep patterns, and mental health outcomes. By integrating theoretical frameworks from disciplines such as psychology, neuroscience, and public health, researchers can gain a holistic understanding of the multifaceted nature of insomnia among college students. This comprehensive approach will not only contribute to theoretical advancements in the field but also inform the development of evidence-based interventions to address this pressing public health issue.

## 5. Limitations and conclusions

To the best of our knowledge, this study is the first bibliometric and visualization analysis of insomnia among college students. There are several limitations in our study. For example, due to the limitations of the software itself, only English-language literature from the WoS core collection was analyzed, and related literature in other languages and other databases was not included, which may have led to some deviations in the results. Therefore, future studies can limit the quality of the included studies.

In summary, VOSviewer 1.6.19 and CiteSpace 6.2. R4 is a practical information visualization software program that can intuitively discover the information hidden in a large amount of research data and its correlation with the help of atlases and reveal the research status and research hotspots of insomnia among college students. At present, there are still several shortcomings in this field: Research teams and institutions should strengthen academic exchanges and cooperation and need to strengthen international cooperation to jointly improve the overall strength of scientific research in this field. In-depth study of the specific mechanisms of insomnia among college students to provide a reference and basis for related research in this field.

## Author contributions

**Conceptualization:** Mengdie Yang, Lingling Li.

**Funding acquisition:** Mengdie Yang.

**Methodology:** Lingling Li.

**Software:** Mengdie Yang, Lingling Li.

**Supervision:** Lingling Li.

**Validation:** Lingling Li.

**Writing – original draft:** Mengdie Yang.

**Writing – review & editing:** Lingling Li.
